# Service Members Prefer a Psychotherapist Who Is a Veteran

**DOI:** 10.3389/fpsyg.2018.01068

**Published:** 2018-06-29

**Authors:** Travon S. Johnson, Alexis Ganz, Stephen Berger, Anindita Ganguly, Gilly Koritzky

**Affiliations:** Clinical Psychology Program, American School of Professional Psychology, Argosy University, Orange County, Orange, CA, United States

**Keywords:** military, mental health, therapy, attitudes, culture

## Abstract

The military is experiencing high rates of mental illness, yet service members and veterans with mental health problems often choose not to seek treatment. Based on clinical-psychology models of client-therapist matching and cultural competency, we hypothesized that willingness to seek treatment among military personnel is higher when the potential psychotherapist is a discharged veteran. Seventy-seven military personnel (73% men, 70% White, *M*_age_ = 34.2) took part in the study. As hypothesized, the majority of participants indicated that they would prefer to see a psychologist who is a veteran. When responding to vignettes, ratings of the psychotherapist’s ability to understand the client (a soldier post-deployment), of his ability to help such a client, and of whether the client should seek treatment from this psychotherapist were higher when the psychotherapist was a veteran compared to when he had no military experience. There were no between-group differences in age, years of service, deployment history, or attitudes toward psychotherapy in general. Similarly, gender and education level had no effect on the results. These findings imply that having the opportunity to receive treatment by a psychotherapist who is a veteran may remove barriers for treatment and encourage more service members and veterans to seek and obtain the help that they need. This can be done by communicating these findings to the military population and by encouraging therapists who have military experience to make this fact known to their potential clients.

## Introduction

The military has been experiencing pervasive levels of mental health problems, including depressive disorders, Post-traumatic Stress Disorder (PTSD), drug and alcohol abuse, and suicide (Hoge et al., 2004; Kim et al., 2010; Black and Collier, 2014; Summerall, 2016). Despite the prevalence of these conditions, most service members with mental health problems prefer not to seek treatment (Kim et al., 2011). This is unfortunate, because psychological therapy has been shown to reduce symptoms for veterans suffering from PTSD and other disorders related to their service (Rauch et al., 2009; Macdonald et al., 2011; Liverant et al., 2012).

A major barrier to seeking treatment is negative attitudes and stigma against mental illness and mental health help (Kim et al., 2011; Draplski et al., 2013; Schreiber and McEnany, 2015). Despite recent campaigns by the Department of Defense to reduce it, stigma of mental health care is a significant problem in the military (Miggantz, 2013). Military personnel may believe that they are expected to handle their psychological distress on their own (Pietrzak et al., 2009), or that seeking psychological help might label them as unfit for service and have bad implications for their future (Stecker et al., 2007). These findings suggest that service members will be reluctant to seek mental health help within the military system, but they may be willing to consider a therapist who is not a member of the military. By seeking a non-military psychologist, they may assume that their mental health issues will not be shared with anyone but the therapist.

However, using the services of a non-military psychologist may also have perceived setbacks in the eyes of service members and veterans. They often feel that civilians who have not experienced combat are unable to understand what they have gone through (Brewin et al., 2011; Ahern et al., 2015; Stein and Tuval-Mashiach, 2015).

A civilian psychologist without military experience may be limited in his or her ability to help a veteran patient, due to lack of familiarity with the military lifestyle, and more profoundly, lack of cultural competence. The military has a unique culture, which includes values, beliefs, and norms of behavior (Danish and Antonides, 2009). Those who serve (or have served) in the military are socialized into this culture, while “outsiders” often find it hard to understand (Reger et al., 2008). As a result, a psychologist or therapist from outside the military culture might be perceived as incapable of helping (Reger et al., 2008). Indeed, culture has been acknowledged as an important facet of the civil-military gap (Rahbek-Clemmensen et al., 2012). One important aspect of this issue is language. A service member’s description of their experiences in the military involves heavy use of jargon, technical terms, and acronyms. The communication between the therapist and the client might be difficult if the client is frequently asked to stop and explain what they meant, and such communication difficulties will get in the way of establishing trust and building a therapeutic alliance.

On the other hand, if the therapist is familiar with the military culture and has knowledge of combat-related terms and procedures, this should facilitate communication and rapport building (or at least, the service members may believe that this is the case). Service members may be more willing to seek therapy from a therapist who has endured experiences (such as deployment) similar to what they endured. They may feel that such a therapist will have a deep understanding of their difficulties, and perhaps will be more willing to speak and self-disclose in their presence. Therefore, in the present study we examine service members’ willingness to seek help from a therapist who is a discharged veteran. Such a therapist has no professional reporting duties to the military system, which should alleviate concerns of stigma and negative implications on one’s career. Nonetheless, the therapist is a member of the military culture with a background similar to that of the patient. Thus, service members’ knowledge of the psychologist’s being a veteran may increase their willingness to seek professional care. Interestingly, although this hypothesis has been previously proposed (Danish and Antonides, 2009), it has not been subjected to an empirical investigation. Nonetheless, several studies have highlighted the importance of peer-support and reliance on peers’ assistance in the military (Greden et al., 2010; Caddick et al., 2015). The implicit rationale is that the “helper” being a part of the military culture facilitates the service member in being receptive to the help offered. Our hypothesis is grounded in theory that emphasizes the importance of a strong rapport between the client and the therapist (Lambert, 1992), which along with confidentiality would be considered a cornerstone of all psychotherapy. Clients should feel a sense of connection and trust with their therapist, and they should believe that their therapist can help them. This relationship, sometimes called *therapeutic alliance*, is considered key in a variety of therapies regardless of the theoretical orientation of the therapist (Cashdan, 1989; Lambert, 1992; Gelso and Carter, 1994; Leibert and Dunne-Bryant, 2015). Numerous studies have suggested that similarity between the client and therapist is positively linked to therapy success, presumably because such similarity helps in building a strong client-therapist rapport, and also facilitates the client feeling understood by the therapist. For instance, Weisman and Tishby (2014) found such an effect on therapy outcome when the client and therapist have similar attachment styles. It has also been found that client-patient similarity is associated with clients’ positive attitudes about the therapy process. This has been observed with respect to client-therapist similarities in gender (Landes et al., 2013) race or ethnicity (Tien and Johnson, 1985; Cabral and Smith, 2011; Landes et al., 2013), faith (Greenidge and Baker, 2012), age and seniority (Lasky and Salomone, 1977), and culture (Dean, 2001; Schim and Doorenbos, 2010). It seems that the client’s comfort with the therapist during the early stages is drastically affected by how much the client identifies with the therapist in relation to themselves. This suggests that service members would be more willing to seek psychological care if their therapist is also a member of the military culture.

Therefore, we hypothesized that when the psychotherapist being considered is a veteran, ratings of the therapist’s ability to understand the military-member client, of the therapist’s ability to help such a client, and of whether the client should seek treatment from this psychotherapist will all be higher than when the psychotherapist has no military experience.

## Materials and Methods

### Participants

The inclusion criteria were: Active duty U.S. military service members or veterans of Operation Iraqi Freedom (OIF) and/or Operation Enduring Freedom (OEF), over 18 years of age. Participants were recruited using an ad that was posted on the Facebook group pages for the Military Police Regimental Association (MPRA) and the Wounded Warrior Project (these pages are frequented by service members and veterans). The ads included an invitation to partake in the study, a list of the inclusion criteria, and a link to the study itself on Qualtrics.com (a software for data collection and analysis, which serves as a platform for running studies online). Ninety-three persons opted to participate in the study by clicking on the link. Of those, a total of 77 provided complete responses and were included in the final sample.

#### Attitudes About Veteran Versus Non-veteran Psychotherapists

We created a vignette describing a soldier who experiences PTSD symptoms following deployment and who considers psychological treatment. The vignette had two versions that were identical except for one sentence, which described the psychologist being considered. In version A the psychologist was also a veteran, and in version B he was not a veteran. The vignette read as follows (the two versions of the psychologist’s description appear in brackets as “version A” and “version B”): “*You have just returned from a 9-month deployment to Afghanistan with Michael, a fellow soldier. You and Michael were a part of an infantry unit who were on foot-patrols 5 days a week. Throughout the deployment, the unit encountered two improvised explosive devises (IEDs) during patrols, one brief firefight, and three mortar attacks on the Forward Operation Base (FOB). It has now been 6 months since returning from deployment and you have noticed that Michael is easily startled to loud and unexpected noises, and is constantly late to formation as he rarely sleeps. He is easily irritated, getting angry and upset frequently, and has been involved in two fights. As a result of the fights, he had been given disciplinary action from his supervisor. You have also noticed that his alcohol use is much higher than everyone else in the unit. Michael has told you that he has trouble falling asleep at night and that he often has nightmares. He also has trouble concentrating during the day. You have noticed that Michael’s behavior was not like this before the deployment. You hear that there is a psychologist, Dr. Nicholas Sheppard, who has an office about 30 min. from the base and keeps all information confidential. He has been in practice for 10 years*. [Version A: *Before becoming a psychologist, he served in the army for 4 years as infantry, and deployed to Iraq in 2003*] [Version B: *In addition, he also teaches college classes in psychology*]. *Dr. Sheppard specializes in treating soldiers and veterans*.”

After reading the vignette the participants were asked to indicate their level of agreement with a list of items on a 1–7 Likert scale: (1) Dr. Sheppard can understand what Michael is going through; (2) Michael needs professional help; (3) I think Michael should see Dr. Sheppard; (4) Michael can solve his problems by himself; (5) Dr. Sheppard is capable of helping Michael.

Items 2 and 4 aimed to assess whether the respondents agreed that “Michael” needs professional help given his circumstances. Items 1, 3, and 5 were designed to measure between-versions differences in how “Dr. Sheppard” is perceived.

#### Participants’ Attitudes Toward Psychotherapy in General

We used the Attitudes Toward Seeking Professional Psychological Help, short form (ATSPPH-SF; Fischer and Farina, 1995). It is a 10-item, 4-point Likert scale, in which higher scores indicate more positive attitudes toward seeking professional help. We used this questionnaire in order to account for individual differences in attitudes toward seeking psychotherapy, which might confound the effect of the psychologist’s veteran status, as measured by the vignettes. Sample items are “*I would want to get psychological help if I were worried or upset for a long period of time*” and “*Personal and emotional troubles, like many things, tend to work out by themselves*” (reverse coded).

In addition to the ATSPPH-SF items, participants were also requested to respond to two items: “I would prefer a psychologist who is a veteran” and “Seeking mental health would hinder my career.” Though separate from the ATSPPH-SF, these items were presented on a similar 4-point Likert scale. Their purpose was to obtain information on issues pertaining to psychological help that are unique to the military population.

#### Demographic Questionnaire

Asked participants to report their age, gender, race/ethnicity, education level, branch of service, rank, status, years of service, type of military job (combat arms, combat support, etc.), whether they had deployed to a combat zone, whether they had experienced combat-related exposure (e.g., small-arms fire, IEDs, mortar attacks, or suicide bombers), and whether they had sought treatment for mental health concerns, either before or since joining the military.

### Procedure

As an incentive to participate, the recruitment post stated that $5 will be donated to the Wounded Warrior Project for every complete response. Study materials – informed consent, the vignettes and questionnaires, and a debriefing statement – were administered through Qualtrics. The assignment to psychotherapist’s status (veteran or non-veteran; version A or B of vignette, respectively) was done randomly by the Qualtrics software. The study was approved by the University’s Institutional Review Board.

### Statistical Analysis

Comparisons between the two groups (versions of the vignette) were done using independent samples *t*-tests, Chi-square tests, or Mann-Whitney *U*-tests, as appropriate for each variable. Further analyses included one-way and two-way ANOVA. Analyses were done using IBM SPSS Statistics 23 software.

## Results

### Sample Demographics and Characteristics of Military Service

The sample was 73% male. The age range was 21–70 years (mean = 34.2, *SD* = 10.03). The most common race/ethnicity category was Caucasian (70%), followed by Hispanic/Latino (14%), African American (5%), Asian/Pacific Islander (5%), and Other (5%). This race/ethnicity distribution is consistent with the Department of Defense reports of the military population (Department of Defense, 2015).

The participants had 2–32 years of military service (mean = 8.96, *SD* = 5.63). Most participants (69%) were honorable discharge veterans, 19% were active duty service members, 9% were Reserves or National Guard, and 2% were general discharge. Most participants (89%) were serving or have served in the U.S. Army, 8% were in the Marine Corps, and 3% were in the Air Force. The most common ranks were junior enlisted non-commissioned officers (E5-E6; 49%), followed by junior enlisted (E1-E4; 30%), senior enlisted non-commissioned officers (E7-E9; 14%), and commissioned officers (O1 and above; 7%). The most common jobs were combat support (military police, combat engineering, intelligence, signal, chemical, etc.; 70%), followed by combat service support (transport, supply, maintenance, medical, chaplain, judge advocate (JAG), etc.; 17%), and combat arms (infantry, artillery, tank drivers, cavalry scout, etc.; 13%). The vast majority (90%) reported that they had deployed to a combat zone, and 76% reported having experienced combat-related exposure. The majority (91%) reported that they had never sought treatment for mental health concerns before joining the military. When asked whether they had sought treatment for mental health concerns since or after joining the military, 58% reported “yes” and 42% reported “no.”

The descriptive statistics of the two groups are given in **Table 1**. As can be seen, the groups were similar in their demographic characteristics except education level. Therefore, we conducted some analyses with education as an additional independent variable (see below).

**Table 1:** Characteristics (means and S.D.) of the Two Study Groups.

	Veteran psychologist (*n* = 39)	Non-veteran psychologist (*n* = 38)	Test statistics	*p*
**Demographics**			
Age	33.7 (8.9)	34.8 (11.2)	*t*_(75)_ = -0.469	0.555
No. years of service	9.4 (6.2)	8.5 (5.1)	*t*_(74)_ = 0.665	0.241
Gender				
% Women	29%	23%	χ^2^_(1)_ = 0.345	0.557
Race/Ethnicity				
% White	66%	77%	χ^2^_(1)_ = 1.169	0.280
Education				
% Has college degree AA or higher	61%	36%	χ^2^_(1)_ = 4.667	0.031^∗^
Status				
% Active duty	18%	21%	χ^2^_(3)_ = 0.054	0.997
% Reserves/National Guard	8%	7%		
% Honorable discharge	71%	69%		
% General discharge	3%	3%		
Branch of service				
% US Army	90%	90%	χ^2^_(2)_ = 2.669	0.263
% US Marine Corps	5%	10%		
% US Air Force	5%	-		
Rank				
% Junior enlisted (E1-E4)	29%	38%	χ^2^_(2)_ = 2.246	0.523
% NCO^b^ (E5-E6)	55%	52%		
% Senior enlisted NCO^b^ (E7-E9)	13%	5%		
% Commissioned officer (O1 & up)	3%	5%		
Military job				
% Combat Arms	8%	17%	χ^2^_(2)_ = 1.400	0.497
% Combat Support	72%	69%		
% Combat Service Support	20%	14%		
% Deployed to a combat zone	87%	92%	χ2_(1)_ = 0.618	0.432
% Experienced combat-related exposure	68%	82%	χ2_(1)_ = 1.924	0.165
% Sought treatment for mental health concerns before joining the military	8%	10%	χ2_(1)_ = 0.130	0.719
% Sought treatment for mental health concerns since or after joining the military	55%	62%	χ2_(1)_ = 0.312	0.576
**Attitudes about Psychotherapy (vignette)**			
Dr. Sheppard can understand what Michael is going through (1–7)	5.61 (1.20)	3.44 (1.71)	*t*_(75)_ = 6.423	<0.001^∗^
Michael needs professional help (1–7)	6.32 (0.96)	6.23 (1.04)	*t*_(75)_ = 0.373	0.710
I think Michael should see Dr. Sheppard (1–7)	5.87 (1.21)	5.10 (1.60)	*t*_(75)_ = 2.361	0.021^∗^
Michael can solve his problems by himself (1–7)	2.05 (1.25)	2.08 (1.18)	*t*_(75)_ = -0.088	0.930
Dr. Sheppard is capable of helping Michael (1–7)	5.53 (1.22)	4.85 (1.44)	*t*_(75)_ = 2.228	0.029^∗^
**Attitudes about Psychotherapy (general)**			
I would prefer a psychologist who is a veteran (1–4)	2.66 (0.78)	2.72 (0.51)	Mann-Whitney *U* = 719.00	0.761
Seeking mental health would hinder my career (1–4)	1.32 (1.02)	0.95 (0.99)	Mann-Whitney *U* = 589.50	0.107
ATSPPH-SF (0–30)^a^	17.50 (5.65)	18.18 (6.02)	*t*_(75)_ = -0.510	0.611

*^a^ATSPPH-SF: Attitudes Toward Seeking Professional Psychological Help, short form. ^b^NCO: Non-commissioned officer. ^∗^Significant difference*.

There were no between-group differences in the respondents’ attitudes about psychotherapy in general, as indicated by the ATSPPH-SF and the two additional items “I would prefer a psychologist who is a veteran” and “Seeking mental health would hinder my career” (see **Table 1**).

As for the vignettes, respondents in both groups assessed the soldier’s condition as equally severe. There was high agreement with the item “Michael needs professional help” and disagreement that “Michael can solve his problems by himself.” However – as hypothesized – responses were significantly more favorable to the soldier being treated by a veteran psychologist than to him being treated by a non-veteran psychologist (see **Table 1**). Of particular interest is the between-group difference in assessing how well the psychologist would be able to understand what the soldier-client is going through. Those who considered a psychologist who is a veteran rated his ability to understand “Michael” as very high, while those who considered a non-veteran psychologist (with the same credentials, level of experience, and expertise) opted for the mid-point of the scale. These results confirm our hypotheses.

Importantly, when asked directly about their own preference, the majority of respondents indicated that they would prefer a veteran psychologist for themselves (77% “agree,” 18% “partly agree,” 2.5% “partly disagree,” and 2.5% “disagree”). This finding further supports our hypothesis.

To account for the potential effect of the difference in education level on the main findings, we conducted a series of two-way ANOVA with psychologist’s veteran status and having a college degree as the independent variables, and the five attitude questions that followed the vignette about the soldier and the potential psychologist as dependent variables. Neither education nor the interaction between education and psychologist’s veteran status were significant factors in any of the models (*p*-values 0.414 or higher).

As can be seen in **Table 1**, the gender distribution was not different between the two conditions. However, due to the overall proportion of women in the sample being higher than found in the military population (Department of Defense, 2015), we conducted a similar series of two-way ANOVA with psychologist’s veteran status and gender as the independent variables. Neither gender nor the interaction between gender and psychologist’s veteran status were significant in any of the models (*p*-values 0.107 or higher).

The respondents indicated some concern that seeking mental health help would hinder their military career (10% “agree,” 27% “partly agree,” 27% “partly disagree,” 10% “disagree”). Although their degree of concern was not high, this is potentially a point in favor of a therapist who is not working for the military at the time the therapy is provided (whether or not he or she has military background). One might conjecture that such concerns would be higher among those who are still in the service than among those who are discharged. However, a one-way ANOVA did not detect any differences in responses as a function of participants’ status with the military (*F*_(3,73)_ = 0.219, *p* = 0.883).

## Discussion

Consistent with our hypotheses, ratings of the psychotherapist’s ability to understand the client (a soldier post-deployment), of his ability to help such a client, and of whether the client should seek treatment from this psychotherapist were higher when the psychotherapist being considered was a veteran compared to when the psychotherapist had no military experience. There were no between-group differences in age, race/ethnicity, characteristics of military service, deployment history, or attitudes about psychotherapy in general. Similarly, gender and education level had no effect on the results.

Furthermore, we found that willingness to seek mental-health treatment among military members and veterans is higher when the potential psychotherapist is a discharged veteran. The majority of participants indicated that they would prefer to see a psychologist who is a veteran.

Although barriers to help-seeking are diverse (Stecker et al., 2007; Pietrzak et al., 2009; Kim et al., 2011; Draplski et al., 2013; Miggantz, 2013; Schreiber and McEnany, 2015), our findings suggest that one such barrier may be overcome by referring service members and veterans to psychotherapists who have served in the military. This is evidenced by the question that was presented directly (“I would prefer a psychologist who is a veteran”) as well as by the participants’ responses to the vignette. It appears that psychotherapists who have served in the military are preferred by this population, especially when it comes to their perceived ability to understand the difficulties that service members are going through.

Our findings are consistent with previous reports that service members and veterans tend to feel misunderstood by civilians (Brewin et al., 2011; Ahern et al., 2015; Stein and Tuval-Mashiach, 2015) and on the importance of peer-support and reliance on peers’ assistance in the military (Greden et al., 2010; Caddick et al., 2015). A potential implication of these findings is that having the opportunity to be seen by a psychotherapist who is a veteran – especially one who is no longer affiliated with the military – may reduce barriers for treatment and encourage more service members and veterans to seek and obtain the help that they need. Potentially, this can be done by communicating these findings to psychologists and the military population alike and by encouraging therapists who have military experience to make this fact known to their potential clients. Furthermore, when such therapists are available, it could be helpful to advertise that information to service members and veterans. In addition, these findings would support the use of military resources to recruit psychotherapists with military experience.

Our findings are consistent with prominent theories of the client-therapist relationship (Cashdan, 1989; Lambert, 1992; Gelso and Carter, 1994; Leibert and Dunne-Bryant, 2015) and models that emphasize matching as a contributing factor in the therapeutic alliance (Tien and Johnson, 1985; Schim and Doorenbos, 2010; Cabral and Smith, 2011; Greenidge and Baker, 2012; Landes et al., 2013; Weisman and Tishby, 2014). Our findings are also consistent with the notion of cultural competency, which holds that therapists and mental health providers should possess cultural knowledge and skills of a particular culture to deliver effective interventions to members of that culture (Dean, 2001; Sue, 2006; Bernal et al., 2009). However, since our findings only apply to attitudes about the therapist’s competency – not actual therapy outcomes – we acknowledge that this interpretation should be made with caution. For instance, other prominent models argue for a universalistic approach (Hampden-Turner and Trompenaars, 2000), which suggests that matching on cultural variables does not necessarily lead to better therapy outcomes. However, it remains an empirical question as to whether findings in regard to other cultural variables generalize to the military population.

Of relevance to the argument for cultural competency is the fact that culture is often discussed as a major feature of the *civil-military gap*, which refers to disagreement and divide between the military and civilian sectors on policy issues (Feaver and Kohn, 2001; Hines et al., 2015). For instance, Rahbek-Clemmensen et al. (2012) argue that cultural differences, such as value differences between military and civilian populations, impact the gap and enhance such divide.

To our knowledge, research is limited on whether matching characteristics of the therapist (such as gender, culture, or military experience) play a role in clients’ initial decision to seek treatment from this therapist. Further research is needed to assess whether and how such knowledge can facilitate treatment-seeking in populations who are otherwise reluctant to seek mental help.

One limitation of the present study is that the sample was too small to enable meaningful cross-sectional analyses, for instance, in comparing between those with and without a history of deployment to a combat zone, or with and without exposure to combat. Other factors, such as rank or branch of service may also be of interest in this context, but our ability to analyze them systematically in this sample has been limited as well.

Another potential limitation is that the vignettes were written in third person and thus measure attitudes indirectly. This was done for two reasons. First, to limit the risk of evoking negative emotional responses in respondents who may be experiencing similar symptoms following a deployment; Second, to remove social desirability concerns, such as respondents being reluctant to think of themselves as potentially being in need of psychological treatment. Nevertheless, when we asked the participants directly whether they would prefer a psychologist who is a veteran, the majority answered in the positive. Thus, the preference of psychotherapists with military background is prevalent when assessed both directly and indirectly.

For similar reasons, namely, to avoid the invoking of distress and in light of expectedly high socially desirable responding, we chose not to assess self-stigmatization or psychopathology directly. We recognize that having this information would have enabled a deeper understanding of the potential determinants of preferring veteran psychologists. Nonetheless, we have asked participants about history of seeking mental-health services before and since their joining the military. While few (9%) reported seeking mental-health services before joining the military, the majority (58%) reported seeking mental-health services since or after joining the military. This may indicate the prevalence of mental-health issues, possibly including conditions such as PTSD as well as other severe disorders, in this population. However, seeking therapy may be attributed to many reasons, and so this information is not sufficient. Therefore, this remains a major limitation of the present study, which should be addressed in future research.

In sum, our findings suggest a potential way to remove barriers to mental health help for service members who need them. They imply that for this population, having a shared set of experiences with the therapist is of significant importance in fostering a favorable attitude toward being treated by them. In light of the burden of mental illness on the military, these findings have considerable clinical implications.

## Ethics Statement

This study was carried out in accordance with the recommendations of the Institutional Review Board (IRB) at Argosy University, Orange County with written informed consent from all subjects. All subjects gave written informed consent in accordance with the Declaration of Helsinki. The protocol was approved by the Institutional Review Board (IRB) at Argosy University, Orange County.

## Author Contributions

TJ developed the hypotheses, conducted the study, and wrote the first draft of the manuscript. AlG led the literature review and assisted in validating the vignettes. SB and AnG reviewed the clinical theory, ensured the accuracy and currency of the clinical models presented, and edited the manuscript. GK supervised the research project, conducted some of the statistical analyses, wrote parts of the manuscript, and prepared the manuscript for publication.

## Conflict of Interest Statement

The authors declare that the research was conducted in the absence of any commercial or financial relationships that could be construed as a potential conflict of interest.
